# STK31 Is a Cell-Cycle Regulated Protein That Contributes to the Tumorigenicity of Epithelial Cancer Cells

**DOI:** 10.1371/journal.pone.0093303

**Published:** 2014-03-25

**Authors:** Pao-Lin Kuo, Yung-Ling Huang, Christine Chin-Jung Hsieh, Jenq-Chang Lee, Bo-Wen Lin, Liang-Yi Hung

**Affiliations:** 1 Department of Medical Laboratory Science BioTechnology, College of Medicine, National Cheng Kung University, Tainan, Taiwan; 2 Institute of Bioinformatics and Biosignal Transduction, College of Bioscience and Biotechnology, National Cheng Kung University, Tainan, Taiwan; 3 Center for Infectious Disease and Signal Transduction Research, National Cheng Kung University, Tainan, Taiwan; 4 Department of Obstetrics and Gynecology, National Cheng-Kung University Hospital, Tainan, Taiwan; 5 Department of Surgery, National Cheng-Kung University Hospital, Tainan, Taiwan; 6 Institute for Cancer Biology and Drug Discovery, College of Medical Science and Technology, Taipei Medical University, Taipei, Taiwan; Institute for Research in Biomedicine, Spain

## Abstract

Serine/threonine kinase 31 (STK31) is one of the novel cancer/testis antigens for which its biological functions remain largely unclear. Here, we demonstrate that STK31 is overexpressed in many human colorectal cancer cell lines and tissues. STK31 co-localizes with pericentrin in the centrosomal region throughout all phases of the cell cycle. Interestingly, when cells undergo mitosis, STK31 also localizes to the centromeres, central spindle, and midbody. This localization behavior is similar to that of chromosomal passenger proteins, which are known to be the important players of the spindle assembly checkpoint. The expression of STK31 is cell cycle-dependent through the regulation of a putative D-box near its C-terminal region. Ectopically-expressed STK31-GFP increases cell migration and invasive ability without altering the proliferation rate of cancer cells, whereas the knockdown expression of endogenous STK31 by lentivirus-derived shRNA results in microtubule assembly defects that prolong the duration of mitosis and lead to apoptosis. Taken together, our results suggest that the aberrant expression of STK31 contributes to tumorigenicity in somatic cancer cells. STK31 might therefore act as a potential therapeutic target in human somatic cancers.

## Introduction

Cell division in mammalian cells is regulated by a number of protein kinases that control progression through various phases of the cell cycle. Previous studies indicated that misregulation of cell cycle kinases might result in the unlimited proliferation and aberrant division of cells leading to genomic instability, both of which are the hallmarks of carcinogenesis [1], [2]. Accumulated evidence indicates that mitotic kinases are responsible for protecting cells from chromosome aberrations and aneuploidy. Mitotic kinases like Polo-like kinases (Plks) and Aurora kinases are involved in regulating the centrosome cycle and mitotic spindle formation. Once the bipolar spindle is formed, spindle assembly checkpoint (SAC) proteins such as Bub1 and BubR1 are required to ensure the proper bipolar orientation of sister chromatids and proper connections between kinetochores and spindle microtubules [2]. Alterations in signaling pathways involved in these mitotic kinases can result in an exit from mitosis with a consequently aberrant chromosome number, leading to aneuploidy and eventually cancer [3].

The centrosome has been regarded as an important component in animal cell division. It is composed of two centrioles with an orthogonal arrangement surrounded by electron-dense pericentriolar material (PCM) [4], [5]. Many centrosomal proteins located in the PCM have been discovered, and they play important roles that are highly correlated with centrosomal functions [6]. These centrosomal proteins can be divided into different classes according to their functions. The first class comprises proteins that serve as scaffolds for the assembly of other proteins, and thus are required for maintaining the structure of the centrosome. There is also a group of proteins that function in microtubule nucleation. Lastly, many regulatory molecules, including kinases, phosphatases and signaling molecules, are implicated in cell cycle regulation [7]. Several lines of evidence indicate that the aberrant expression of centrosomal proteins or centrosome dysfunction can be linked to tumorigenesis [8]–[10].

Targeting the mitotic kinases has been considered a highly successful strategy for anticancer treatment [11], [12]. Small molecular inhibitors targeting CDKs, Aurora kinases, or Plks have been investigated for their ability to interrupt the development of cancer [13]–[16]. These inhibitory compounds show efficacy *in vivo* on human tumor xenografts, and some of them are currently being investigated in clinical trials [17]. On the other hand, cancer therapy involving immunotherapy using T-cells, which recognize cancer antigens, has become a promising cancer treatment approach [18], [19]. Thus, the identification of novel tumor antigens and tumor-specific T-cell epitopes is helpful in cancer immunotherapy. One group of tumor antigens is called the cancer/testis antigens (CTAs), its expression normally being limited to the testis [20]. Immunogenic cancer vaccines targeting CTAs do not pose a significant risk of adverse events because their expression are restricted to male germ cells, an immunologically privileged site of body, and thus are ideal targets for treating cancer.

Our previous report indicated that the *serine/threonine kinase 31* (*STK31*) mRNA level in testicular tissues of patients without mature germ cells is significantly lower than that of normal men [21]. A previous report showed that STK31 is a novel CTA [22]. Here, we investigate the involvement and physiological role of STK31 in cancer development. We found that STK31 localizes to the centrosome throughout all stages of the cell cycle. During mitosis, STK31 shares similar subcellular localizations with the mitotic kinases Aurora-B and Plk1. The cell cycle-dependent expression of STK31 is controlled by ubiquitin-proteasome degradation. Depletion of STK31 affected the microtubule assembly process during interphase, suggesting its potential role in microtubule nucleation. In addition, a deficiency of STK31 delays mitotic progression, results in a failure to exit from mitosis, and leads to apoptosis. Taken together, our study provides a potential cancer therapeutic strategy that can be considered for the future applications.

## Materials and Methods

### Patient Specimens

Human colorectal cancer tissues were obtained in accordance with the Declaration of Helsinki. The study was approved by the Institutional Review Board of the National Cheng Kung University Hospital. The written informed consent from the donor was stored in the National Cheng Kung University Hospital database and used for research.

### Plasmids, Cell Lines and Transfection

Human STK31 full length cDNA was cloned into vector pEGFP-N1. AZ521, a human colorectal cancer cell line, was cultured in DMEM medium (Sigma) plus 10% MEM (GIBCO) and 1% NEAA (Bio-West). HCT116, a human colorectal cancer cell line, was maintained in RPMI-1640 medium (GIBCO). All media were supplemented with 10% FBS (Bioscience), 100 units/ml penicillin, and 100 μg/ml streptomycin (GIBCO). For transient transfection, cells were grown up to 80% confluence and transiently transfected with STK31-GFP using Lipofectamine 2000 (Invitrogen) according to the manufacturer’s protocol.

### Lentiviral Short-hairpin RNA (shRNA) Transfection

The pLKO.1 Mission short RNA (shRNA) vectors against *STK31* (TRCN0000003274, TRCN0000003275, TRCN0000003276, and TRCN0000028838-A4, TRCN0000028841-B4, TRCN0000028758-F3, TRCN0000028817-G1, TRCN0000028819-H2) were obtained from National RNAi Core Facility (Institute of Molecular Biology/Genomic Research Center, Academia Sinica, Taiwan). The efficiency of *STK31* shRNA was monitored by both Immunoblot and Q-PCR analysis (Figures S2A and S2B). For STK31 knockdown, cells were grown up to 80% confluence and infected with *STK31* shRNAs by a multiplicity of infection (MOI) of four supplemented with 8 μg/ml polybrene (SIGMA). After 48 h of infection, 1 μg/ml puromycin (SIGMA) was added to the growth medium for selection. After an additional 48 h of culture, cells were collected for total RNA purification or total cell lysate extraction.

### Western Blot Analysis and Antibodies

Cell lysates were prepared in lysis buffer (150 mM NaCl, 1% NP40, 0.5% Na-deoxycholate, 1 mM EDTA, and 50 mM Tris-HCl; pH 7.4) with the addition of the 1X protease inhibitor cocktail (Roche Molecular Diagnostics) and 1X phosphatase inhibitor cocktail (P0044, Sigma). Anti-α-tubulin (DM1A), anti-γ-tubulin (GTU88), anti-actin, anti-GAPDH, anti-cyclin B, anti-phospho-Histone 3/serine 10, and anti-Aurora-B antibodies were purchased from Sigma. Anti-myc antibody was obtained from Invitrogen. Anti-pericentrin (Ab4448) was purchased from Abcam. Anti-GFP antibody (JL-8) was purchased from Clontech.

### Real Time PCR and Primers

Total RNA was extracted using Trizol reagent (Invitrogen) according to the manufacturer’s illustration. Reverse transcription was performed with 1 μg of total RNA using Improm-II Reverse Transcriptase (Promega). Real-time PCR was performed using the SYBR advantage qPCR premix (Bio-Rad) in a CFX96 Real-Time System and C1000 Thermal Cycler (BIO-RAD), and the reaction was performed using the following conditions for 44 cycles: 95°C for 15 s, 60°C for 10 s, and 72°C for 5 s. The sequences of primers used are as follows: forward primer and reverse primer for human *STK31* are 5′-CAGGACCAGAAACTGATTGAAG-3′ and 5′-TCCATTCAAAGAAGCTGGAGTAG-3′, respectively. Real-time fluorescence monitoring and melting curve analysis were performed with Bio-Rad according to the manufacturer’s recommendations (Bio-Rad). Data were analyzed by Bio-Rad CFX Manager software version 1.5 to determine the threshold cycle (*Cp*) above background for each reaction. The relative amount of the target gene was normalized to that of *actin* of the same cDNA.

### Immunofluorescence Staining

Cells grown on coverslips were rinsed with PBS and then fixed with 3.7% formaldehyde for 6 min at room temperature, or fixed by ice-cold methanol/acetone (w/w, 1∶1) for 10 min at −20°C. After fixation, cells were permeabilized by incubating with 0.01% Tween-20 in PBS for 3–5 min at room temperature. To detect centrosomes or microtubules, cells were stained with monoclonal anti-γ-tubulin (GTU-88, 1∶200 dilution) or anti-α-tubulin (DM1A, 1∶200 dilution), respectively, followed by incubation with Alexa Fluor 488 or 568 rabbit anti-mouse IgG (1∶200 dilution, Invitrogen). To detect endogenous STK31, cells were stained with monoclonal mouse STK31 antibody, 1G10, followed by incubation with Alexa Fluor 488 or 568 rabbit anti-mouse IgG. Images were observed with a fluorescence microscope (Personal DV Applied Precision, Issaquah, WA) having a deconvolution function (*softWORX*).

### Flow Cytometry

The AZ521 cells with different cell cycle processing were fixed with 80% ethanol for 24 h at −20°C and then stained with propidium iodide (PI) for 30 min at room temperature. Cell cycle phase distribution was analyzed by an FACS Calibur instrument (BD Biosciences).

### TUNEL Assay

Cells were fixed with 4% paraformaldehyde and permeabilized by 0.1% Triton X-100 in PBS supplied with 0.1% sodium citrate. The TUNEL assay was performed with an *In Situ* Cell Death Detection Kit, TMR, (Roche) according to manufacturer’s protocol.

### Wound-healing Assay

AZ521 cells and HCT116 cells were seeded into 3 cm dishes with a concentration of 1×10^5^ cells per well. After 24 h of transfection with GFP and GFP-STK31, cells were wounded by gentle tip-scratching. The width of the lesion area ranged from 500 μm to 1 mm and was imaged by microscope at 0 h, 6 h, 12 h, and 24 h. Migration efficiency was measured by wound interval.

### Transwell Assay

A transwell with a pore size of 3.0 μm (Millipore, Bedford, MA) was coated with 5 μg/cm^2^ collagen gel and air dried overnight or 0.5 mg/ml matrigel (BD biosciences) for 1 h. GFP- or GFP-STK31-transfected cells were resuspended in culture media without 10% fetal bovine serum (FBS) and seeded into a transwell placed in a 24-well plate containing growth medium with 10% FBS. After 24 h incubation, the transwell was rinsed with PBS and fixed with 3.7% formaldehyde for 10 min at room temperature. Finally, filters were removed from the wells and mounted with ProLong Gold antifade reagent with DAPI, and the cells on the filters were counted under an immunofluorescence microscope (Olympus, BX51).

## Results

### Expression of STK31 in Various Human Cancer Cell Lines and Clinical Human Colorectal Tissues

**Figure 1:**
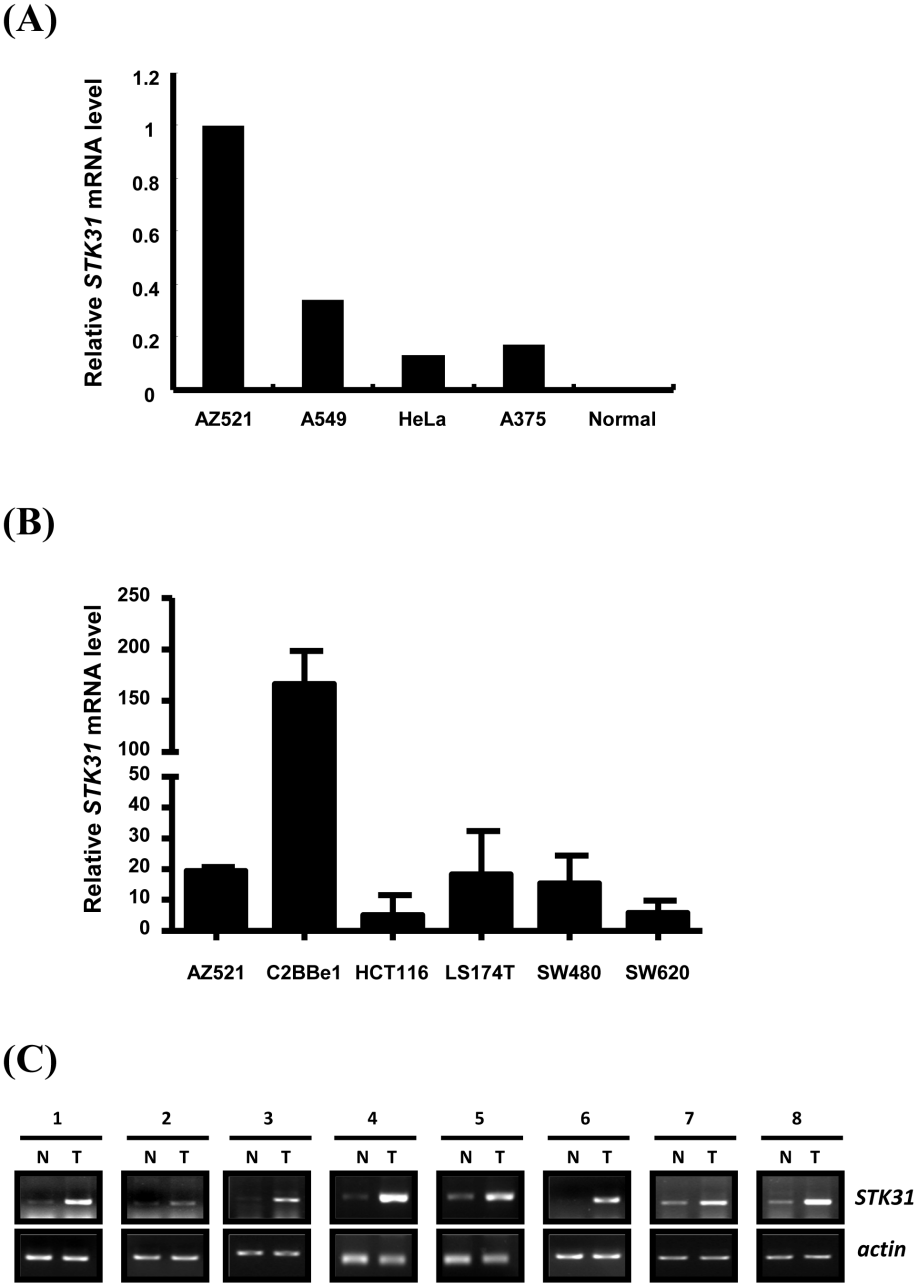
Expressions of *STK31* in human cancer cell lines and colorectal cancer. (**A**) Total RNA from four human cancer cell lines of different tissues, AZ521 (a human colorectal cancer cell line), A549 (a human lung cancer cell line), HeLa (a human cervical cancer cell line), A375 (a human melanoma cancer cell line), and one normal human mammary epithelial cell (Normal) that was isolated for real-time PCR to determine *STK31* mRNA levels. *STK31* was significantly overexpressed in the human gastric cancer cell line, AZ521, compared to other human cancer cell lines. (**B**) Total RNA prepared from six gastrointestinal cancer cell lines were used to detect *STK31* mRNA expression level by real-time PCR as described in A. (**C**) Paired human colorectal cancer tissues (N, normal tissue; T, tumor tissue) were collected to isolate total RNA for detecting the expression levels of *STK31* by RT-PCR. *Actin* was used as an internal control. Eight representative paired specimens were shown.

*STK31* overexpression in clinical colorectal cancer specimens has previously been reported [23]. To analyze the expression of *STK31* in cancer cell lines, Q-PCR was performed on human cancer cell lines AZ521, A549, HeLa, and A375. Among these, which originated from different tissues, AZ521, a gastric adenocarcinoma, showed the highest *STK31* mRNA expression (Figure 1A). The expression of *STK31* mRNA was further evaluated in many colorectal cancer cells compared with AZ521. The results showed that all of those cells expressed high levels of *STK31* (Figure 1B). The same result occurred in human clinical colorectal tissues, which expressed high levels of *STK31* in cancerous tissues relative to normal tissues from the same person (Figure 1C). Based on the high level of endogenous *STK31* mRNA, AZ521 was used as the cell model in this study.

### Endogenous STK31 is Located in the Centrosome, Kinetochore, Central Spindle, and Midbody during Mitosis

**Figure 2:**
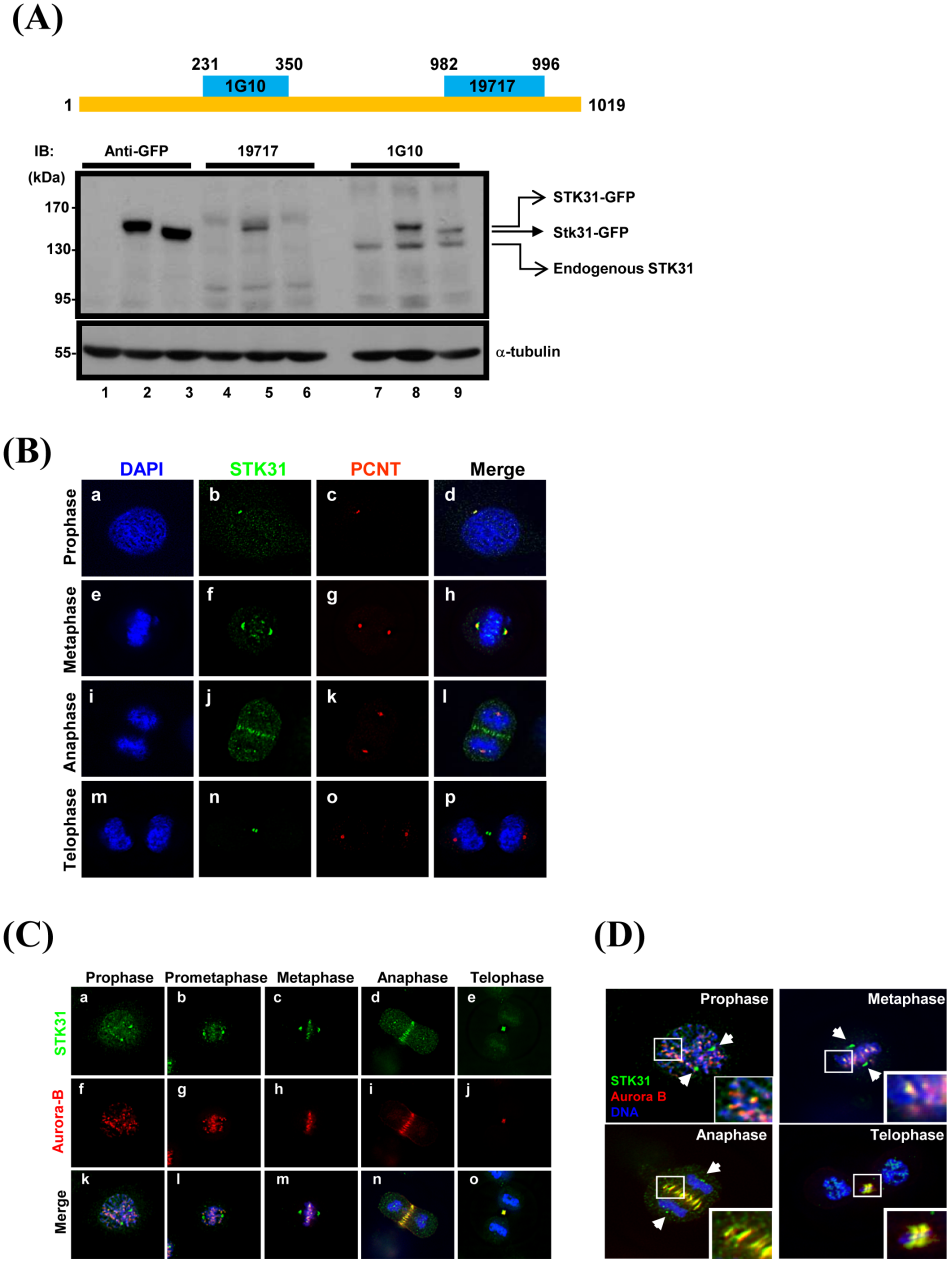
Subcellular localization of STK31. (**A**) Schematic representation of the STK31 polypeptide. Two epitopes, amino acids 231–350 and 982–996, were used as antigens to generate the monoclonal antibody, 1G10, and polyclonal antibodies, 19717, respectively. After purification, the specificity of 19717 (lanes 4–6) and 1G10 (lanes 7–9) was confirmed by immunoblot (IB) analysis using AZ521 total lysate (lanes 1, 4, and 7), STK31-GFP (contains the human form STK31 lanes 2, 5, and 8), or Stk31-GFP (contains the mouse form Stk31 lanes 3, 6, and 9) transfected AZ521 cell lysates. Anti-GFP was used as a positive control for IB to detect the expression of STK31-GFP and Stk31-GFP (lanes 1–3). α-tubulin was used as a loading control. (**B**) AZ521 cells were doubly stained with 1G10 (green) and pericentrin (red, a known centrosomal protein). DNA was visualized using DAPI (blue). Prophase, a–d; metaphase, e–h; anaphase, i–l; and telophase, m–p. Merged images are shown on the right (d, h, l, and p). (**C**) AZ521 cells were co-immunostained with 1G10 (green, a–e) and anti-Aurora B (red, f–j) antibodies. The merged images are shown (k–o). (**D**) Cells were doubly stained with STK31 (green), Aurora-B (red), and DNA (blue). Only merged images were shown. Arrows indicated the colocalization of STK31 and Aurora-B throughout the cell cycle.

In order to further investigate the physiological roles of STK31 in somatic cancer cells, specific STK31 monoclonal (1G10, Figure 2A) and polyclonal (19717) antibodies were generated against different human STK31 peptides (Figure 2A, upper). The specificity of these two antibodies was demonstrated by Western blot analysis of total lysates from AZ521 cells exogenously expressing EGFP-tagged human STK31 protein (STK31-GFP) or mouse Stk31 protein (Stk31-GFP) (Figure 2A). The results show that endogenous STK31 (Figure 2A, lane 7), exogenous STK31-GFP (Figure 2A, lane 8), and Stk31-GFP fusion protein (Figure 2A, lane 9) can be detected by the monoclonal antibody, 1G10. Polyclonal STK31 antibodies, 19717, can only detect the exogenous STK31-GFP (Figure 2A, lane 5), but not the endogenous STK31 and Stk31-GFP (Figure 2A, lanes 4 and 6). The specificity of the monoclonal antibody, 1G10, on A549 cells and AZ521 cells was also confirmed to detect the endogenous STK31 (Figure S1). The differential protein levels of A549 cells and AZ521 cells are in line with mRNA expression as determined by real-time reverse transcription PCR (Figure 1A).

To gain insight into the biological function of STK31 in cancer cells, the subcellular localization of endogenous STK31 was determined. AZ521 cells were co-immunostained with antibodies against STK31 (1G10) and pericentrin (PCNT), a centrosome-associated protein by immunofluorescence analysis. During prophase and metaphase, STK31 co-localized with PCNT, suggesting an association with spindle poles (Figure 2B, a–h). In addition, STK31 was found to reside at the centromeric region during metaphase (Figure 2B, e–h), spindle midzone during anaphase (Figure 2B, i–l), and finally concentrated at the midbody during telophase (Figure 2B, m–p).

To further confirm the localization of STK31 during mitosis, AZ521 cells were co-immunostained with antibodies against STK31 and the chromosomal passenger complex (CPC) protein, Aurora-B (Figure 2C). CPC is composed of Aurora-B kinase, INCENP, surviving, and borealin [24], and is known to migrate from inner centromeres to the spindle midzone and equatorial cell cortex during the metaphase-anaphase transition. Immunofluorescence staining showed that Aurora-B displays the cellular re-localization behavior that has been indicated in previous studies (Figure 2C, f–j). Remarkably, STK31 staining (Figure 2C, a–e) was found to be co-localized with Aurora-B at the centromeric region, spindle zone, and midbody (Figure 2C, k–o). Note that in addition to the co-localization with Aurora-B, STK31 was also found to have strong signals at spindle poles (Figure 2D, arrows). This subcellular distribution of STK31 is similar to that of Polo-like kinase 1 (PLK1) [25].

### The Expression of STK31 is Cell Cycle-dependent

**Figure 3:**
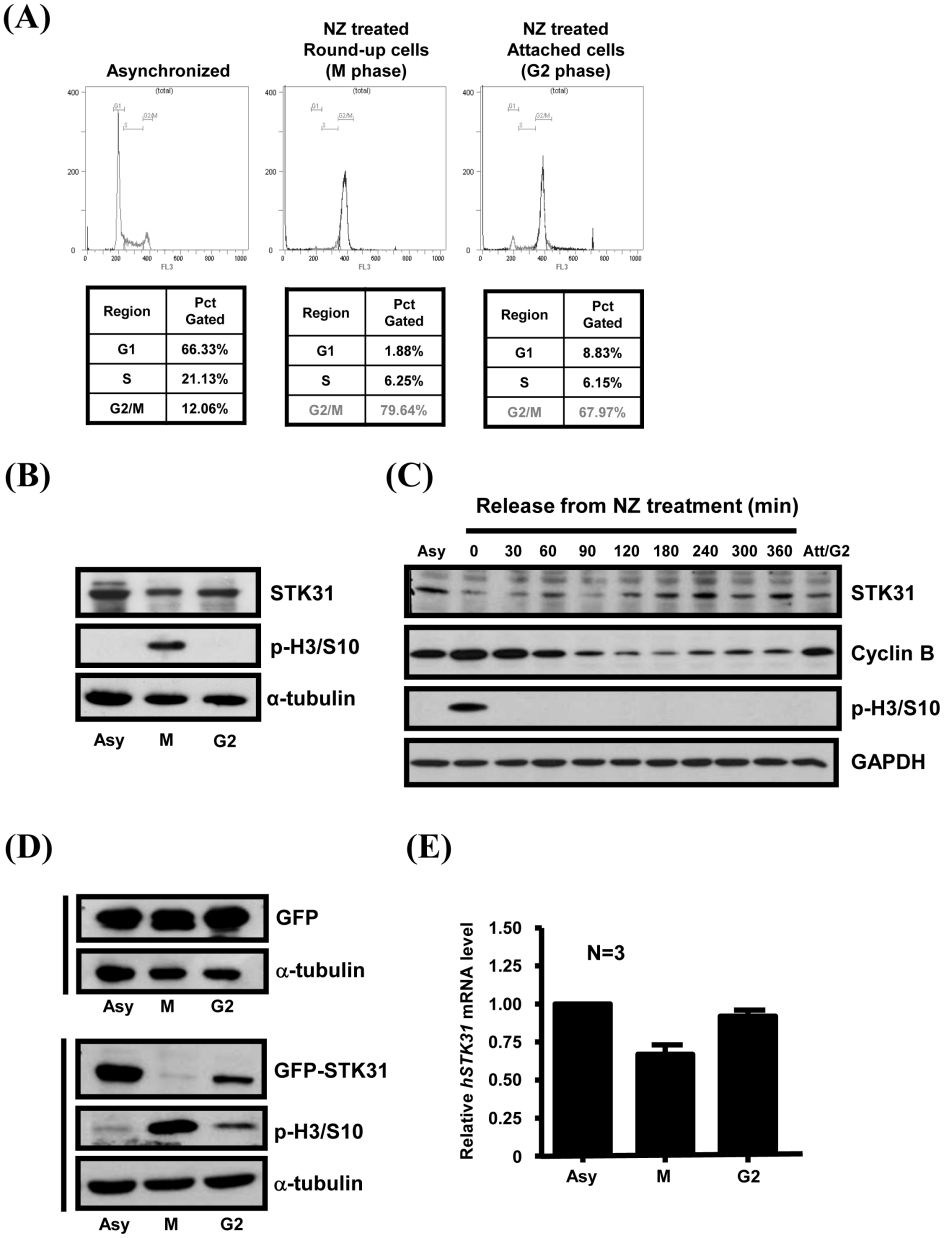
Cell cycle expression pattern of STK31. (**A**) Asynchronized or nocodazole (NZ)-treated AZ521 cells were collected to analyze their cell cycle population by flow cytometry. The percentages of G1, S, and G2/M are shown below. The round-up cells after NZ treatment were shake-off and defined as M phase cells, and the attached cells remaining after shake-off were considered to be the G2 phase. (**B**) Total AZ521 lysates from asynchronized (Asy), M phase (M), and G2 stage of cells were collected for Western blot analysis with anti-STK31 polyclonal antibodies. Phospho-histone H3 serine 10 (P-H3/S10) is a marker for mitotic cells. (**C**) AZ521 cells released from nocodazole treatment with different time points (from 0 to 360 min) were collected to analyze the expression pattern of STK31 by IB analysis. Anti-Cyclin-B and anti-phospho-histone H3 (p-H3/S10) antibodies were used as mitotic markers. (**D**) AZ521 cells ectopically expressing GFP or STK31-GFP were treated with nocodazole. M phase round-up cells were collected by shake-off, and remaining attached cells collected as the G2 phase. Asynchronized cells (Asyn) are shown. α-tubulin or GAPDH was used as the internal control for IB. (**E**) Total RNA from different cell cycle stages of AZ521 was harvested to analyze the expression of *STK31* mRNA by real-time PCR. Three independent experiments were performed (N = 3), and means with standard errors are shown.

**Figure 4:**
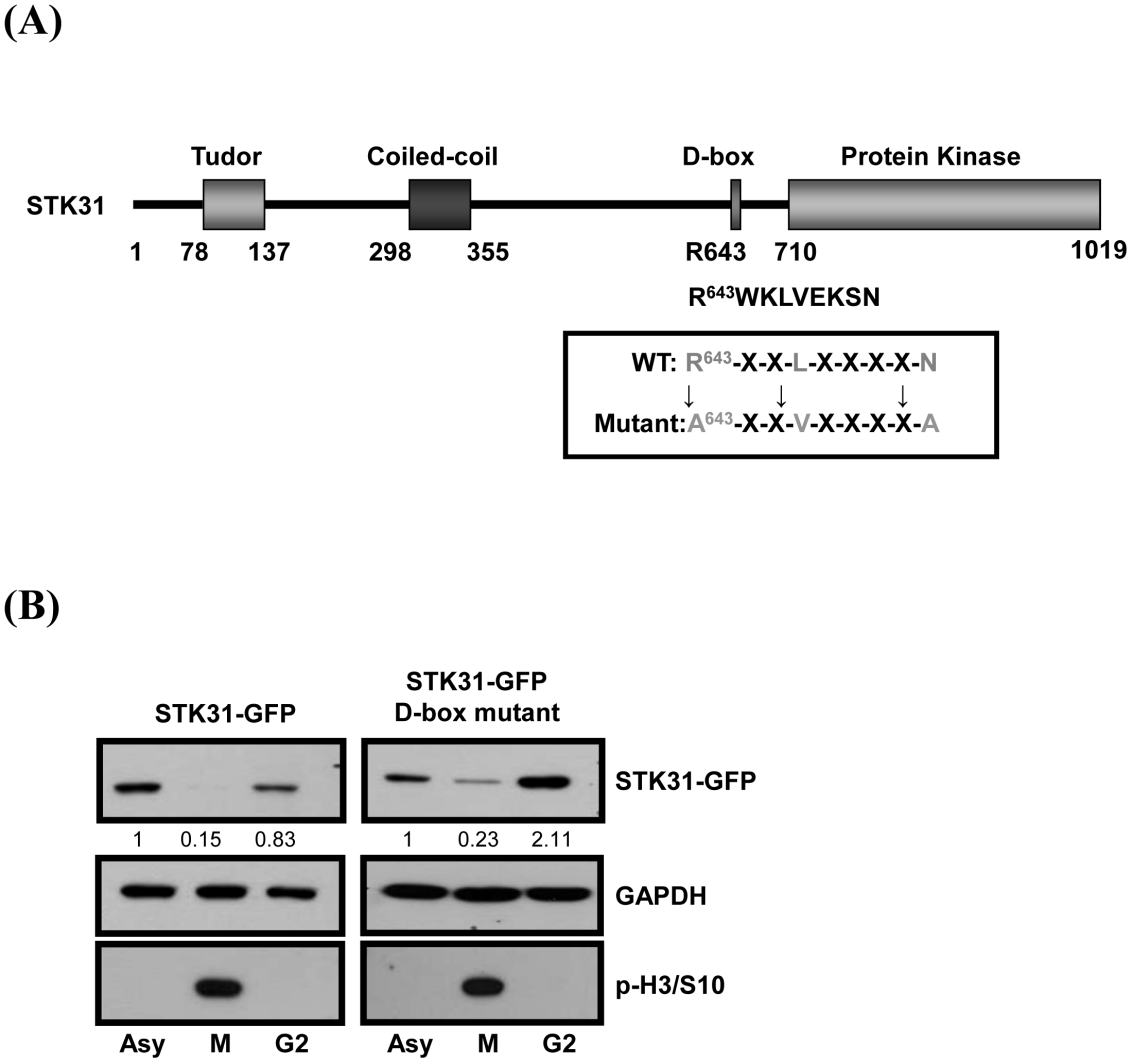
STK31 contains a putative D-box motif. (**A**) A putative D-box motif is located within the amino acids 643–651 of STK31. The conserved position, Arg643 (R643), within the D-box was shown. (**B**) Asynchronized (Asy), M phase, and G2 stage AZ521 cells with STK31-GFP or STK31-GFP/D-box mutant expression were collected to perform the IB analysis using anti-GFP antibody. Expression levels of STK31-GFP are displayed as ratios. Phospho-histone H3 serine 10 (P-H3/S10) is a marker for mitotic cells. GAPDH is a loading control.

To investigate the cell cycle expression pattern of STK31, AZ521 cells synchronized by nocodazole (NZ) or double thymidine treatment were confirmed by flow cytometry (Figure 3A), and the expression of endogenous STK31 protein was determined by Western blot analysis. The result showed that STK31 protein expression is decreased during the G2 stage and reached its lowest level at the M phase (Figure 3B). Western blot analysis from total cell lysates released from nocodazole treatment showed that STK31 protein levels were lowest in NZ-treated cells (Figure 3C, lane 2), and after release, STK31 gradually increased during mitosis (Figure 3C, lanes 3–10). After exiting mitosis, the cell cycle re-entered the G1 stage, which was indicated by Cyclin-B1 degradation at 180 min after release, and the STK31 level increased (Figure 3C, lane 7). Ectopically-expressed STK31-GFP presents the same expression pattern with endogenous STK31 (Figure 3D). Two main strategies are involved in cell cycle-regulated proteins. First, protein abundance can be controlled at the transcription level. Second, protein turnover by proteolytic degradation, which is the main mechanism that regulates the exit from mitosis, is targeted by the APC/C-Cdh1 complex through its destruction box (D-box). We first checked the expression of endogenous *STK31* mRNA by Q-PCR, and found that *STK31* mRNA decreased at prometaphase (Figure 3E). Interestingly, even the protein expression of STK31 is presented at a lower level in the G2 phase (Figure 3B), and the mRNA level has no obvious difference with the asynchronized cells (Figure 3E). In addition, by analyzing the amino acid sequence of STK31, we found that STK31 contains the conserved D-box motif at the Arg^643^ position (R^643^) (Figure 4A). To confirm that the decreased level of STK31 at mitosis exit is due to APC/C-induced ubiquitin-proteasome degradation, the D-box mutant of GFP-STK31 was generated to analyze its cell cycle expression pattern (Figure 4A). From Figure 4B, expression of the STK31-GFP D-box mutant was significantly increased in the G2 phase relative to the wild type STK31-GFP.

### The Overexpression of STK31 Increases Cell Migration and Invasiveness

**Figure 5:**
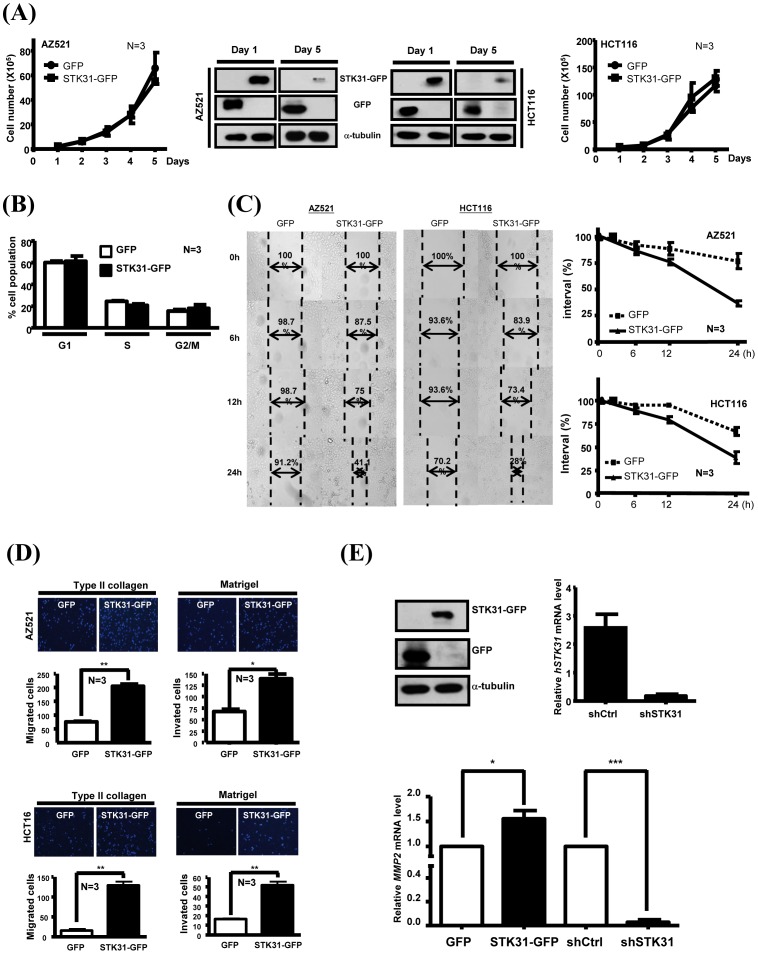
Overexpression of hSTK31 does not promote cell proliferation, but increases cell migration and invasive abilities. (**A**) Equal amount of AZ521 or HCT116 cells with GFP or STK31-GFP expression were seeded, and cell growth was then monitored by calculating viable cell numbers at the indicated time. Three independent experiments were performed and their quantitative results are shown. The expression of GFP or STK31-GFP at Day 1 and Day 5 was detected by IB using anti-GFP antibody. α-tubulin is an internal control. (**B**) AZ521 cells with GFP or STK31-GFP expression were collected to analyze cell population distribution by flow cytometry. (**C**) AZ521 or HCT116 cells with GFP or STK31-GFP expression were used to perform the wound-healing migration assay. The interval of cell migration at different time (0–24 h) was measured and is shown. Quantitative results are shown below. (**D**) AZ521 (upper) or HCT116 (lower) cells with GFP or STK31-GFP expression were seeded to type II collagen-coated or matrigel-coated trans-wells to perform cell migration or invasion assays. After stained with DAPI, a DNA specific dye, cells were counted and the quantitative results are shown. Three independent experiments were done. (**E**) Cells transfected with STK31-GFP or infected with lentiviral-based *STK31* shRNA (sh*STK31*) were collected to detect the expressions of *MMP-2* by real-time PCR (right). Overexpressed STK31-GFP was detected by IB analysis (left). The expression of *STK31* in sh*STK31* cells was determined by real-time PCR and normalized by *β-actin* (middle).

Due to the overexpression of STK31 in cancer cell lines and colorectal cancer tissues (Figure 1), the physiological role of STK31 in tumorigenesis was under investigated by ectopically-overexpressed STK31-GFP. STK31-GFP did not increase the cell proliferation rate in AZ521 and HTC116 cells (Figure 5A) or the cell cycle distribution (Figure 5B). However, the overexpression of STK31-GFP did enhance the cell migration ability of AZ521 and HTC116 (Figure 5C). Based on transwell migration and invasion assays, overexpressed STK31-GFP enabled more AZ521 and HTC116 cells to pass through the matrigel or collegen-coated wells (Figure 5D). By Q-PCR analysis, the expression level of *matrix metallopeptidase 2* (*MMP2*), a migration marker, was increased in STK31-GFP-expressed cells but largely depressed in sh*STK31*-transfected cells (Figure 5E).

### The Depletion of STK31 Causes Microtubule Assembly Defects during Interphase

STK31 was postulated to involve microtubule organization on the basis of its centrosomal localization. To test this, we used a cold treatment to induce microtubule depolymerization and then induce microtubule re-growth. Cells were subsequently fixed and immunostained with anti-α-tubulin to observe the re-assembly and elongation of microtubules. Cells with *STK31* shRNA tagged with EGFP (sh*STK31*-GFP, the knockdown efficiency please see Figure S2B) showed defects on three levels: little/no defects (Figure S3A), moderate defects (Figure S3B) and severe defects (Figure S3C). We compared the cells transfected with sh*STK31*-GFP and the cells transfected with Luciferase shRNA tagged with EGFP (shLuc-*GFP*) as controls, and determined the number of cells for each type (Figure S3D). The results showed that the cells depleted in STK31 failed to show proper organization of microtubules.

### The Knockdown Expression of STK31 by shRNA Results in Mitotic Progression Delay in the GC-1 Cell

To analyze how the depletion of STK31 affects mitotic progression, we used live-cell time-lapse microscopy to monitor the fate of GC-1 cells transfected with sh*STK31*-GFP and compared them with cells transfected with sh*Luc*-GFP (Figure S4) after released from a double thymidine block. We timed the duration of mitotic progression from chromosome condensation (mitotic entry) to cytokinesis (mitotic exit). We found that it took 185 min for the sh*Luc*-GFP cell to complete mitosis (Figures S4A–S4H), whereas 357 min are required for the sh*STK31*-GFP cell to reach telophase (Figures S4I–S4P). The duration of the interval of cells transfected with sh*STK31*-GFP increased two-fold compared to transfection with sh*Luc*-GFP (Figure S4, P and H, respectively). This suggests that the STK31 deficiency causes a delay in mitotic progression in the GC-1 cell.

### The Knockdown Expression of STK31 by shRNA Causes Mitotic Catastrophe and Leads to Apoptosis in Cancer Cells

**Figure 6:**
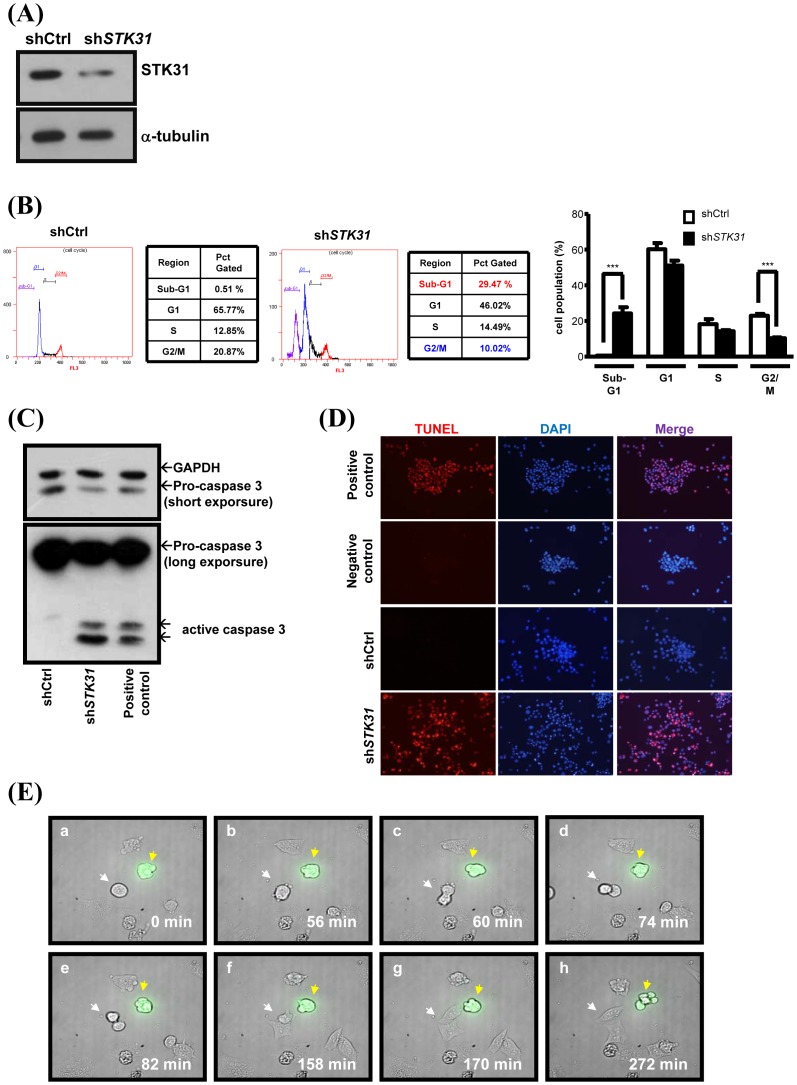
Knockdown expression of hSTK31 results in cell apoptosis. (**A**) After 72 h infection of lentiviral-based *STK31* shRNA (sh*STK31*) or control shRNA (shCtrl), AZ521 cells were harvested to determine the STK31 expression by IB. (**B–D**) Control (shCtrl) or sh*STK31*-infected cells (sh*STK31*) were collected to analyze the cell cycle population by flow cytometry (B), to detect the expression of active caspase-3 by IB (C), and to perform the TUNEL assay (D). Cells treated with DNase I were used as positive controls in C and D. Cells without treatment were the negative controls for the TUNEL assay. The red signal in 6D represents the apoptotic cells. ***, *P* value <0.001. (**E**) Time-lapse images of cells transfected with *STK31* shRNA tagged with EGFP (sh*STK31*-GFP) (A–H). The cells were synchronized with nocodazole (150 ng/ml) for 16 h. After release from nocodazole, cells were observed with time-lapse microscopy. Neighboring untransfected cells (indicated by the white arrow) successfully underwent mitosis and complete cytokinesis, whereas the knockdown cell (indicated by the yellow arrow) remained at a prolonged mitotic-like stage. We timed the duration of mitosis from nuclear envelope breakdown (at 0 min, a) and found that at 272 min the knockdown cell ended up with an apoptotic-like feature (h).

We then investigated the potential role of STK31 using the knockdown approach. Western blotting was performed on AZ521 cells infected with lentiviral STK31 shRNA (sh*STK31*) obtained from Academia Sinica to test knockdown efficiency (Figure 6A). We first checked the effect of lentiviral sh*STK31*-infected cells interfering with the cell population due to subcellular localization and the cell cycle-dependent expression pattern of STK31. Flow cytometry analysis indicated that an increased subG1 and a decreased G2/M were observed in lentiviral sh*STK31* infected cells (Figure 6B). To confirm whether STK31 deficiency leads to apoptosis, both active caspase 3 and a TUNEL assay were carried out on AZ521 cells infected with lentiviral sh*STK31*. From the Figures 6C and 6D, STK31 knockdown cells showed an obvious apoptosis population.

We further asked whether depletion of STK31 induces the same effect on mitotic progression in cancer cells. Likewise, time-lapse microscopy was used to monitor AZ521 cells transfected with sh*STK31*-GFP. Cells were treated with nocodazole for 16 h and then underwent live-cell imaging. The sh*STK31*-GFP cells failed to exit from mitosis, while the control cells successfully completed mitosis and entered the G1 phase (Figure 6E). Note that the sh*STK31*-GFP cell exhibited a mitotic catastrophe and subsequently ended up with an apoptotic cell-like structure (Figure 6E). These observations suggest that STK31 knockdown cells failed to complete mitosis and triggered apoptosis.

## Discussion

*Stk31* was initially identified as a potential sterile gene by cDNA subtraction of mouse spermatoginia [26]. By using cDNA microarray to analyze gene expression in testicular tissues of men with spermatogenic defects, *STK31* was identified as one of the novel genes that may be involved in human spermatogenesis [21]. Recently, STK31 was found to be a cancer-testis antigen (CTA) overexpressed in colorectal cancers [22]. *STK31* mutations were also identified by targeted deep sequencing of kinomes in gastric cancers [27]. These findings shed light on the potential roles of STK31 in cancer. In the current study, real time PCR showed a differential expression pattern of *STK31* in various human cancer cell lines. In addition, *STK31* showed the highest expression level in a gastric cancer cell line, AZ521 (Figure 1A). This finding is consistent with the previous finding that STK31 is overexpressed in clinical specimens of gastrointestinal cancers [22].

To gain insights into the physiological role of STK31 in cancer cells, we examined the subcellular localization of STK31 (Figure 2). Localizations of STK31 (i.e. centromeric localization during metaphase, displacement to the spindle midzone during anaphase, and finally concentrating at the midbody during telophase) are reminiscent of the dynamic localization of chromosomal passenger complex (CPC) proteins. The CPC proteins are composed of Aurora-B, INCENP, Survivin, and Borealin. Aurora-B, the enzymatically active member of CPC, functions in the regulation of chromosome interactions with microtubules, chromatid cohesion, spindle stability and cytokinesis [28]. Among these processes, the spindle assembly checkpoint (SAC) is by far the most important mechanism by which cells prevent abnormal chromosome segregation and aneuploidy. The SAC signaling pathway is known to ensure proper connections between kinetochores and spindle microtubules [29]. Defects in SAC can result in either mitotic catastrophe that triggers apoptosis or an exit from mitosis with aberrant chromosomal distribution [29]. In this study, the depletion of STK31 by RNAi resulted in mitotic catastrophe and apoptosis (Figure 6). Hence, it is tempting to hypothesize that STK31 is closely associated with the SAC pathway. On the other hand, our analysis of the subcellular localization of STK31 also showed that STK31 remained associated with the centrosome throughout all the stages of cell cycle (Figure 2). The centrosome is known as the microtubule organizing center. Its major function is to nucleate microtubules and to orchestrate the cytoskeleton of the cell. A cold treatment experiment showed that STK31 knockdown caused microtubule assembly defects (Figure S3), suggesting that STK31 is involved in microtubule organization and probably acts as a scaffold protein because of its coiled-coil domain (Figure 4A).

We are the first group to demonstrate the expression of STK31 is cell cycle-dependent (Figure 3). Most cell cycle regulated proteins expression is controlled by the anaphase-promoting complex/cyclosome (APC/C) [30]. The activation of APC/C depends on the interaction with Cdc20 or Cdh1, which control substrate specificity through its association with the destruction-box (D-box) or KEN box [31]. The degradation of those substrates displays differential timings during cell cycle progression. Nek2A and cyclin A are degraded at the onset of nuclear envelope breakdown [32], [33], whereas cyclin B1 and securin are degraded after correcting the microtubule-kinetochore attachment [34]. Cells coordinate those events in order to advance the cell cycle smoothly. Our results suggest that the increased expression of STK31 before the mitosis entry (G2 stage) may be mediated through the regulation of a putative D-box located near the C-terminal region (Figure 4). However, more evidence is needed to prove that STK31 is a *bona fide* APC/C substrate, and the detailed molecular mechanism for cell cycle dependent expression pattern of STK31 is still unclear.

Human STK31 is expressed in spermatogonia and spermatocytes [35], [36], and mouse Stk31 has been shown to be expressed in the meiosis-related genes in the mouse fetal ovary [37]. Our preliminary results show that Stk31 localizes to the centrosomal region in mouse spermatogonial cells (GC-1 cell line, data not shown). This suggests that STK31 might play an important role in the regulation of cell cycle progression during male germ cell development. In gametogenesis, mechanisms sensing abnormalities and checkpoint controls are critical for preventing aneuploidy in the embryo derived by aneuploid sperm and oocytes, which is the leading cause of spontaneous abortions and pregnancy loss in human [38]. Further studies are needed to explore the role of STK31 in human reproduction.

In the study reported by Kim Lam Fok *et al.*, STK31 knockdown results in significant suppression of tumorigenicity in colon cancer cells, and STK31 overexpression helps maintain an undifferentiated status of colon cancer cells [23]. However, the signaling pathways have been unclear. The present study shows that an overexpression of STK31 increases cell migration and invasiveness (Figure 5), while the knockdown expression of STK31 causes mitotic catastrophe and apoptosis in cancer cells (Figure 6). These phenotypes may be mediated through the interfering SAC and/or the centrosome functions, given the expression of STK31 in both pathways. Taken together, this report demonstrates a novel CTA that may be involved in cell cycle regulation. We have also shown herein that depletion of STK31 induces apoptosis. STK31 might serve as a potential target for cancer treatment given that inhibitory compounds targeting cell cycle kinases such as Polo-like kinase 1, Aurora-A and Aurora-B have been quite successful in treating cancers [12], [15], [39].

## Supporting Information

Figure S1 **Characterization of STK31 monoclonal antibody, 1G10.**
(TIF)

Figure S2 **Knockdown efficiency of STK31 shRNA.**
(TIF)

Figure S3 **STK31 knockdown results in microtubule assembly defect at interphase.**
(TIF)

Figure S4 **STK31 knockdown causes a delay in mitotic progression in GC-1 cells.**
(TIF)
