# Supplementary figures and images for: STK31 Is a Cell-Cycle Regulated Protein That Contributes to the Tumorigenicity of Epithelial Cancer Cells
Source: PLoS One. 2014 Mar 25;9(3):e93303. doi: 10.1371/journal.pone.0093303 (PMC3965560; doi:10.1371/journal.pone.0093303)

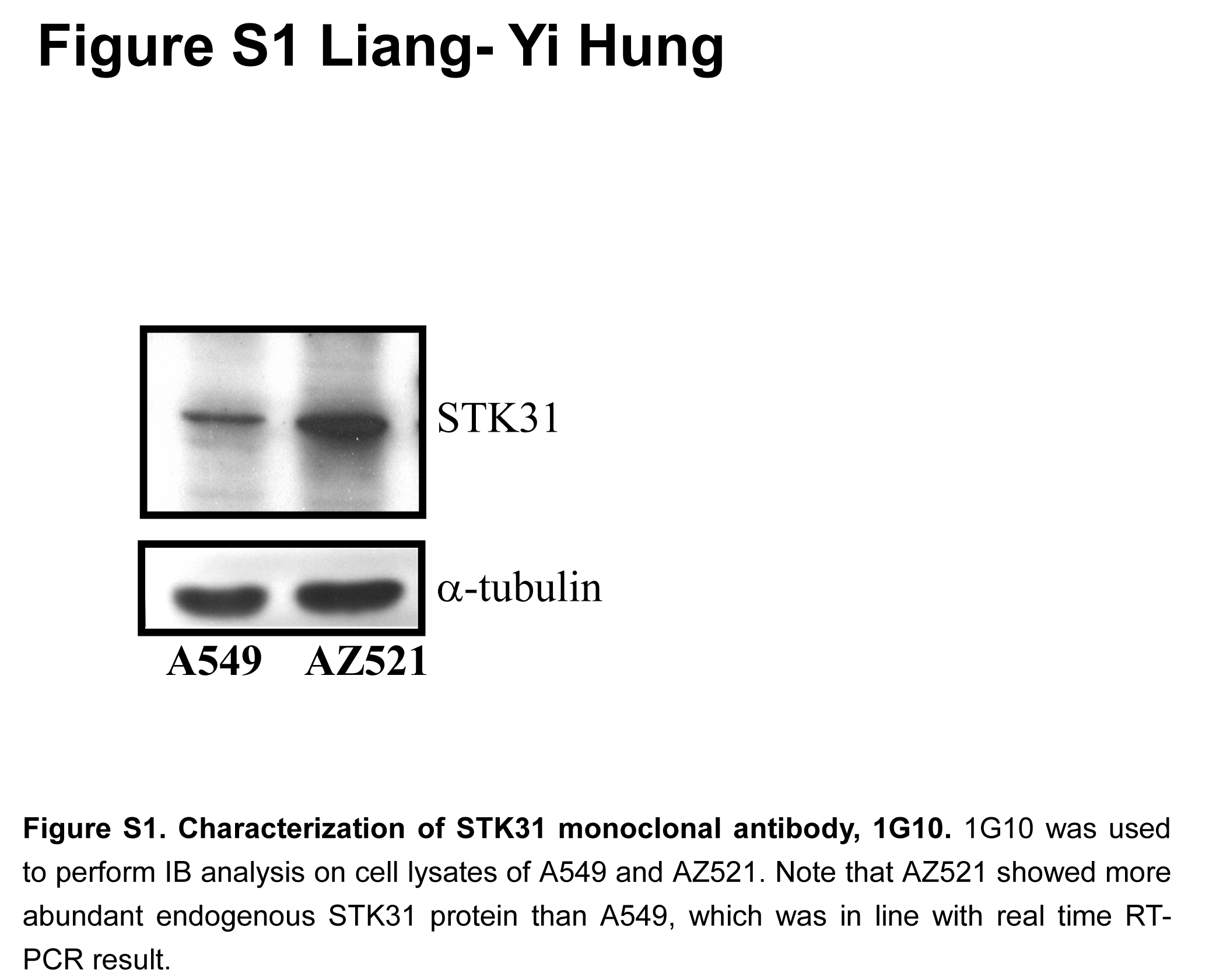

Supplement: Figure S1 — Characterization of STK31 monoclonal antibody, 1G10. (TIF) [file pone.0093303.s001.tif]

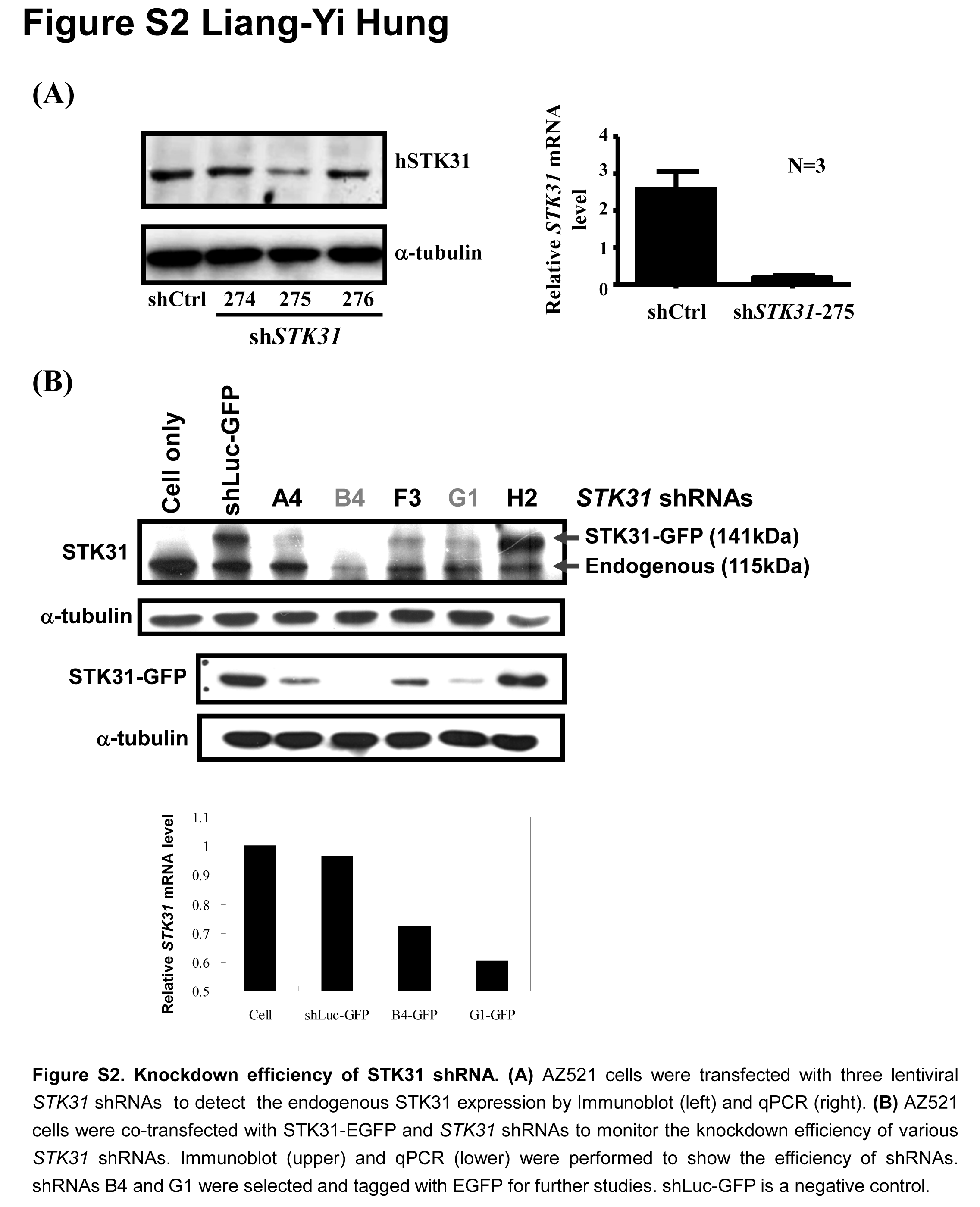

Supplement: Figure S2 — Knockdown efficiency of STK31 shRNA. (TIF) [file pone.0093303.s002.tif]

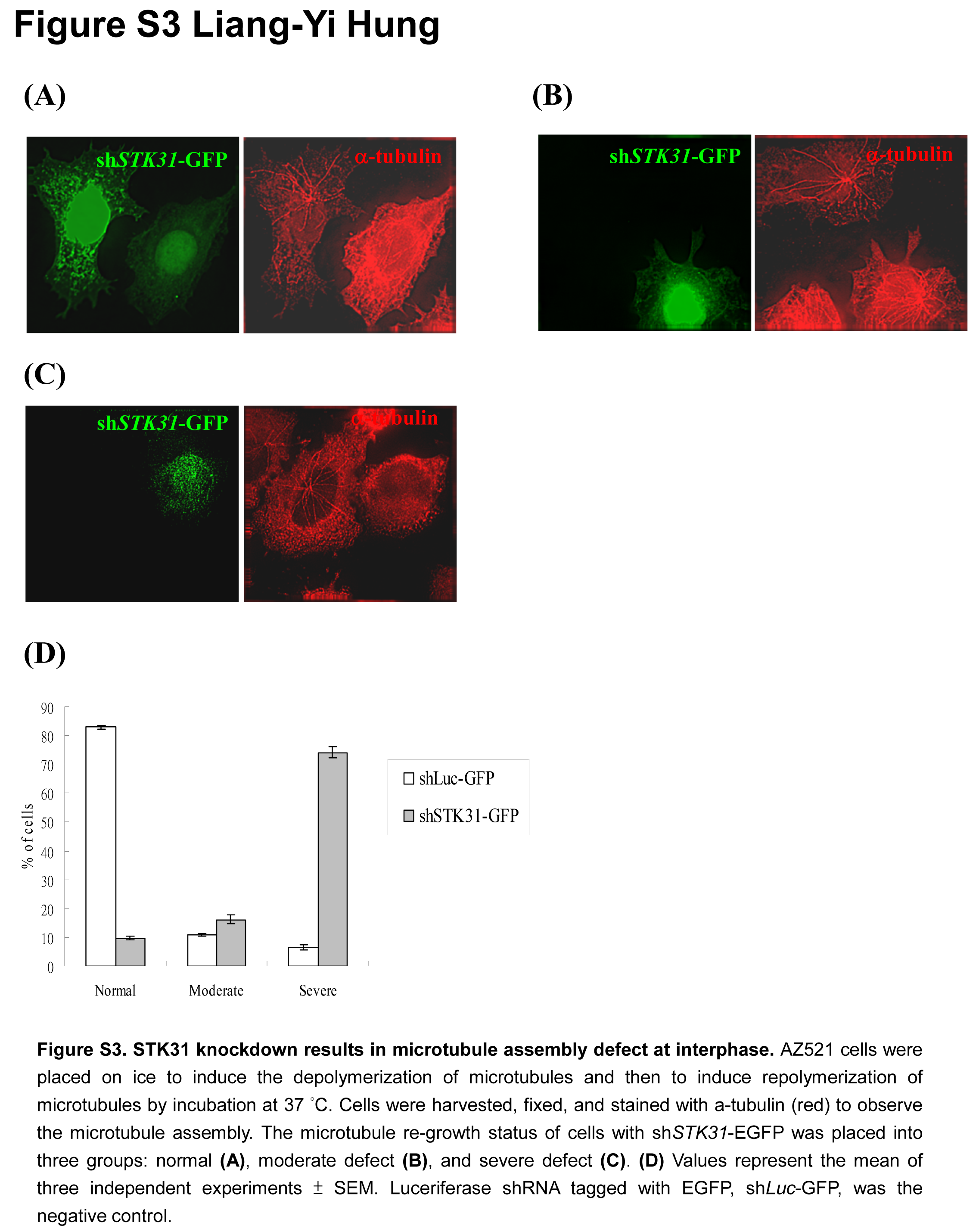

Supplement: Figure S3 — STK31 knockdown results in microtubule assembly defect at interphase. (TIF) [file pone.0093303.s003.tif]

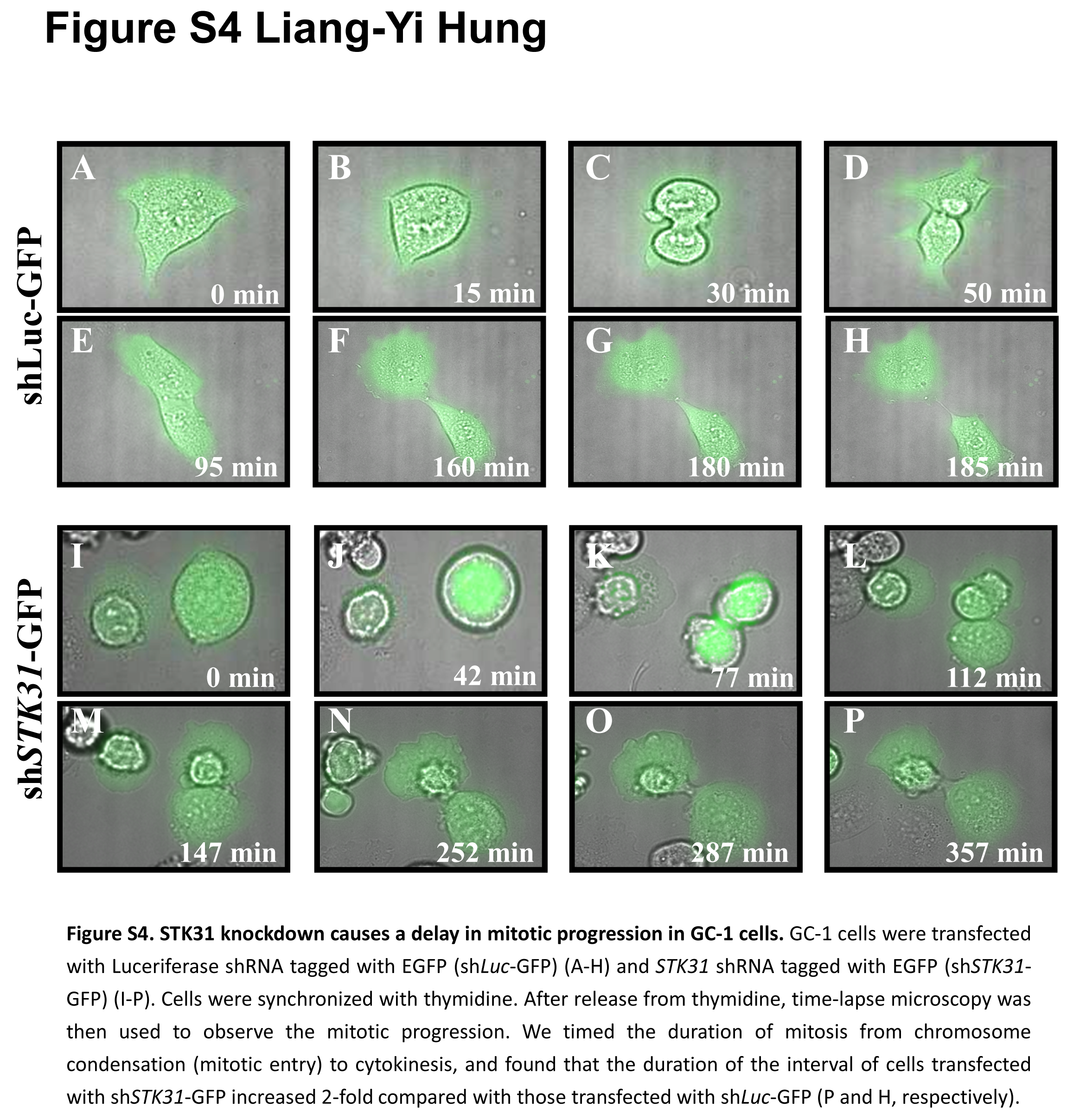

Supplement: Figure S4 — STK31 knockdown causes a delay in mitotic progression in GC-1 cells. (TIF) [file pone.0093303.s004.tif]
